# Pulmonary Hypertension in Adults With a Systemic Right Ventricle (Biventricular Circulation)

**DOI:** 10.1016/j.jacadv.2026.102974

**Published:** 2026-07-02

**Authors:** William M. Wilson, Nael Aldweib, David S. Celermajer, Alexander R. Opotowsky, Payam Dehghani, Jolien Roos-Hesselink, Mikael Dellborg, David Baker, Jamil Aboulhosn, Petra Antonova, Susan Fernandes, Salil Ginde, Frank Han, Clare O’Donnell, Carla P. Rodriguez-Monserrate, Flavia Fusco, Anitha S. John, Joshua Wong, Elizabeth Yeung, Berardo Sarubbi, Stephen Pylypchuk, Sangeeta Shah, Joseph Kay, Jonathan Cramer, Timothy Cotts, Tripti Gupta, Luke J. Burchill, Paul Khairy, Isabelle Vonder Muhll, Alexandra van Dissel, Jasmine Grewal, Anthony Magalski, Pastora Gallego, Fred Rodriguez, Marissa Kuo, Robert M. Kauling, Christopher DeZorzi, Eric V. Krieger, Shelby Kutty, Jeremy Nicolarsen, Craig S. Broberg, Michael Cheung

**Affiliations:** a Department of Medicine, Royal Melbourne Hospital, Melbourne, Australia; b Knight Cardiovascular Institute, Oregon Health and Science University, Portland, Oregon, USA; c University of Sydney and Royal Prince Alfred Hospital, Sydney, Australia; d Cincinnati Children’s Hospital Medical Center, Heart Institute, Department of Pediatrics, University of Cincinnati College of Medicine, Cincinnati, Ohio, USA; e Regina General Hospital, Regina, Saskatchewan, Canada; f Erasmus Medical Center, Rotterdam, the Netherlands; g Institute of Medicine, Sahlgrenska Academy, University of Gothenburg, Gothenburg, Sweden; h UCLA Medical Center, Los Angeles, California, USA; i University Hospital Motol, Prague, Czech Republic; j Departments of Pediatrics and Medicine, Stanford University, School of Medicine, Palo Alto, California, USA; k Children's Hospital of Wisconsin, Milwaukee, Wisconsin, USA; l University of Mississippi Medical Center, Jackson, Mississippi, USA; m Green Lane Paediatric and Congenital Cardiac Service, Auckland City Hospital, Auckland, New Zealand; n Boston Children’s Hospital, and Department of Pediatrics, Harvard Medical School, Boston, Massachusetts, USA; o Division of Cardiology, Department of Medicine, Brigham and Women’s Hospital, Harvard Medical School, Boston, Massachusetts, USA; p Monaldi Hospital, Napoli, Italy; q Children’s National Hospital, Washington, DC, USA; r Colorado University School of Medicine, Denver, Colorado, USA; s University of Alberta, Edmonton, Alberta, Canada; t Ochsner Medical Center, New Orleans, Louisiana, USA; u Children’s Hospital, Omaha & University of Nebraska Medical Center, Omaha, Nebraska, USA; v University of Michigan Medical Center, Ann Arbor, Michigan, USA; w UC San Diego Health, San Diego, California, USA; x Mayo Clinic, Rochester, Minnesota, USA; y Montreal Heart Institute, Montreal, Quebec, Canada; z Amsterdam University Medical Center, Amsterdam, the Netherlands; aa St.Paul’s Hospital, Division of Cardiology, University of British Columbia, Vancouver, British Columbia, Canada; ab University of Missouri- Kansas City and Saint Luke's Hospital, Kansas City, Missouri, USA; ac Hospital Universitario Virgen Del Rocio, Sevilla, Spain; ad Emory University Hospital, Atlanta, Georgia, USA; ae University of Washington Medical Center and Seattle Children's Hospital, Seattle, Washington, USA; af Johns Hopkins University, Baltimore, Maryland, USA; ag Providence Spokane, Spokane, Washington, USA; ah Royal Children's Hospital, Melbourne, Australia

**Keywords:** congenitally corrected transposition of the great arteries, pulmonary hypertension, transposition of the great arteries

## Abstract

**Background:**

Little is known regarding pulmonary hypertension (PH) in adults with a systemic right ventricle.

**Objectives:**

This study evaluates the hemodynamic profile of PH in patients with transposition of great arteries palliated with an atrial switch repair (TGA-AS) and congenitally corrected TGA (CCTGA).

**Methods:**

This was a retrospective cohort study of adults with TGA-AS or CCTGA who had undergone invasive hemodynamic assessment. Exclusion criteria were single ventricle anatomy, previous Rastelli, arterial switch, or double switch operation. PH was defined by a mean pulmonary artery pressure (mPAP) >20 mm Hg and subtypes according to 2022 European Society of Cardiology guidelines. The primary combined clinical endpoint was death, heart transplantation, or need for mechanical circulatory support.

**Results:**

A total of 261 patients were studied (TGA-AS, n = 161 and CCTGA, n = 100). PH prevalence was similar in both groups (65% vs 69%, *P* = 0.74). PH subtype was precapillary in 24%, isolated postcapillary (IpcPH) 25%, combined postcapillary and precapillary 51% (similar in both groups). The relationship between pulmonary capillary wedge pressure and mPAP was overall similar between 2 groups but there was greater variability in pulmonary capillary wedge pressure once mPAP >40 mm Hg in the TGA-AS group. The incidence of the primary endpoint was similar in both groups (24 v 29%; *P* = 0.39). Factors associated with the primary clinical endpoint were elevated PVR (in particular, in the TGA-AS subgroup) and lower aortic pulsatility index (both groups).

**Conclusions:**

In this large study evaluating the hemodynamic phenotype in patients with a systemic RV referred for catheterization, PH was common and most commonly the combined postcapillary and precapillary subtype.

Patients with d-transposition of great arteries (TGA) palliated with an atrial switch procedure (TGA-AS) and congenitally corrected TGA (CCTGA) have a biventricular circulation with a morphologic right ventricle (RV) as the systemic ventricle (2V-RV) and account for a significant proportion of the adult congenital heart disease (ACHD) population.^1^, ^2^, ^3^ Long-term outcomes in each have been reported previously by this research alliance.^4^^,^^5^ Patients with TGA-AS are known to be susceptible to the development of pulmonary hypertension (PH)^2^^,^^6^, ^7^, ^8^, ^9^ but the prevalence in patients with CCTGA is unclear.^2^^,^^10^^,^^11^ The mechanism for PH in the 2V-RV population remains uncertain, in particular whether related to abnormalities of the pulmonary vasculature (especially if late repair of a shunt) or secondary to elevated filling pressures (due to RV dysfunction, tricuspid regurgitation (TR), and noncompliant baffles causing abnormal atrial transport in the TGA-AS group).^2^^,^^6^^,^^7^^,^^9^^,^^12^ PH can occur late after neonatal arterial switch repair, suggesting developmental or genetic causes may be contributory in a subset of TGA-AS patients.^8^ A better understanding of the mechanism for PH in patients with a 2V-RV circulation may afford risk stratification and aid decision-making for ACHD specialists, in particular in the context of advanced heart failure (use of pulmonary vasodilators; use of mechanical circulatory support [MCS]; candidacy for heart or combined heart-lung transplantation).

This study aimed to evaluate the prevalence of and mechanism for PH using invasive hemodynamic data from a large, multicenter registry, whilst comparing findings for TGA-AS and CCTGA.

## Methods

This is a retrospective cohort study undertaken by the Alliance for Adult Research in Congenital Cardiology. It was approved by the Institutional Review Board at Oregon Health Science University and by the research oversight boards at each center. Inclusion criteria were: patients with d-TGA with previous atrial switch procedure or CCTGA; seen at least twice by an ACHD specialist (over more than 12 months); age ≥18 years at time of initial assessment; and had undergone cardiac catheterization. Exclusion criteria were: single ventricle anatomy; prior Rastelli procedure; arterial switch operation; or double switch operation. For patients with CCTGA born with a ventricular septal defect (VSD), valvar, or subvalvar pulmonary stenosis, double outlet RV were considered “complex”.

The first outpatient ACHD encounter since January 1, 2002 was identified as the initial visit. Data from medical records were entered into a secure online database. We recorded anatomic, interventional, and medical history as well as vital signs and medications from the initial visit. We gathered semiquantitative designations for RV systolic function (normal, mild dysfunction, moderate dysfunction, and severe dysfunction) and TR severity (nil, mild, moderate, or severe). From the total cohort, we identified patients who had undergone invasive hemodynamic assessment during the study period and collected data, including: heart rate, systemic blood pressure, superior vena cava, and inferior vena cave pressures, right atrial (RA) pressure, left ventricular (LV) systolic pressure, pulmonary artery (PA) pressure, pulmonary capillary wedge pressure (PCWP), RV end diastolic pressure, and cardiac output (thermodilution used if available, otherwise Fick estimation used). The following were calculated: stroke volume, transpulmonary gradient (TPG), pulmonary vascular resistance (PVR), aortic pulsatility index (API), PA pulsatility index (PAPI), RV stroke work index (RVSWI), LV stroke work index, PA compliance (PAC), PA/systolic blood pressure ratio, diastolic pressure gradient (DPG) and cardiac power. Definitions for these estimated parameters are provided in Supplemental Table 1. Noninvasive brachial blood pressure measurements were used in calculations utilizing aortic pressure due to incomplete collection of central aortic blood pressure measurements.

PH was defined as mean PA pressure (PAP) (mPAP) >20 mm Hg according to the 2022 European Society of Cardiology (ESC) guidelines.^5^ PH, if present, was classified as: precapillary if mPAP >20 mm Hg; PCWP ≤15 mm Hg and PVR >2 WU; combined precapillary and postcapillary PH (CpcPH) if mPAP >20 mm Hg, PCWP >15 mm Hg and PVR >2 WU; isolated postcapillary (IpcPH) if mPAP >20 mm Hg, PCWP >15 mm Hg, and PVR ≤2. Classification of PH type according to the 2015 ESC guidelines^13^ afforded comparison to previous studies (precapillary mPAP ≥25 mm Hg + PCWP ≤15 mm Hg; CpcPH PCWP >15 mm Hg, DPG ≥7 mm Hg and/or PVR >3 WU; IpcPH PCWP >15 mm Hg, DPG <7 mm Hg, and/or PVR ≤3 WU).

The primary endpoint was defined as death from any cause, cardiac transplantation, or need for MCS. Clinical data were collected for the most recent visit and for the most recent clinical visit preceding death, transplantation, or need for MCS (if an event occurred).

### Statistical analysis

Statistical analyses were conducted using SPSS (version 29.0.2.0, IBM 2024) and R software (version 4.4.2, R Core team 2024).

Baseline characteristics and investigation findings were categorized according to diagnosis (TGA-AS vs CCTGA) and according to whether the primary endpoint occurred.

Data underwent internal consistency checks and discrepancies were addressed by local investigators. Only patients with complete hemodynamic data sets were included. Descriptive statistics outlined the characteristics for each group. Comparative analyses utilized the Student’s independent samples *t* test, the chi-square test, the Mann-Whitney U test, or the Fischer exact test for continuous and categorical variables, as appropriate for the data type and whether continuous distributions were consistent with normality. Mean ± SD, median and IQR or n (%) were used to summarize data distributions. Cutoff values for hemodynamic parameters were established using recognized normal thresholds for the following variables: RA pressure>10 mm Hg, PCWP >15 mm Hg, mPAP >20 mm Hg, (and mPAP >40 mm Hg, mPAP >60 mm Hg), TPG >15 mm Hg, DPG >7 mm Hg, and PVR >2 WU (and PVR >5WU). Cutoff values for other variables (eg. indexed PVR, PAC, PAPI, API) were based on the median value and/or receiver operator curves with use of Youden’s index (for prediction of primary endpoint).

Univariable Cox proportional hazard regressions assessed associations between selected variables and the primary outcome. A multivariable Cox regression model for freedom from major adverse cardiovascular events was fitted using selected variables from univariable analyses. Variables were selected to investigate simultaneously the most relevant clinical factors; when different clinical thresholds were considered for a variable (eg, mPAP >20, >40 or >60 mm Hg), the threshold with the highest HR was used. The variable selection avoided using the same input for different predictors (eg, PCWP, PCWP/CI). The proportional hazards assumption was checked for the multivariable model by reviewing whether Schoenfeld residuals were constant over time. A significant deviation from proportional hazards for CCTGA vs TGA-AS existed and was resolved by inclusion of an interaction term, such that the HR for morphology was allowed to differ between 0 to 3 and 3+ years post catheterization. Multicollinearity among predictors was assessed via the variance inflation factor of each predictor (all below 2.0, which is considered low and an acceptable degree of collinearity). All statistical tests were 2-sided and a statistical significance threshold of *P* = 0.05 was adopted.

The Kaplan-Meier curves, stratified by predefined values for various hemodynamic parameters, were calculated to further investigate differential survival between groups and the log-rank test to check for differences in survival between groups. The baseline time was time at first catheter study for both the Cox regression and Kaplan-Meier analyses.

## Results

**Figure 1:**
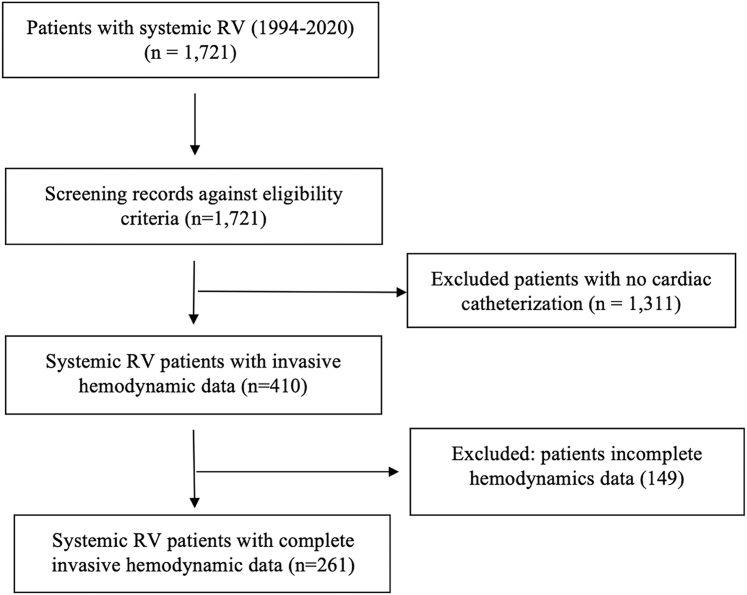
**Patient Selection** RV = right ventricle.

Of the initial 1721 patients included in the registry, 410 (23.8%) underwent cardiac catheterization during the study period. 261 patients (TGA [n = 161] and CCTGA [n = 100]) had complete data for all measured hemodynamic parameters and were included in this analysis (Figure 1). Individuals were followed for a median duration of 5.6 years (4.8-6.8 years) from time of catheterization (TGA 5.2 [3.8-6.0] years vs CCTGA 7.4 [5.2-9.7] years; *P* = 0.03). Cardiac catheterization was performed more frequently in patients with CCTGA than those with TGA-AS (20% vs 15%; *P* < 0.001).

Baseline characteristics of patients who underwent cardiac catheterization vs those who did not are presented in Table 1. Those who underwent catheterization were more likely to reside in the United States (74 vs 51%; *P* < 0.001) (Supplemental Table 2). They tended to have more comorbid diagnoses and more likely to be prescribed cardiac medications. They were more likely to have moderate or severe RV dysfunction and moderate or severe TR. Adverse cardiac events were more frequent in those patients who underwent cardiac catheterization (death/transplant/MCS was 20% vs 6%; *P* < 0.0001).

**Table 1:** Comparison of Baseline Characteristics and Outcomes Between Patients Who Underwent Cardiac Catheterization and Those Who Did Not

	Overall (N = 1,721)	Referred for Catheterization (n = 410)	No Cardiac Catheterization (n = 1,311)	*P* Value
Age at first visit, y	31.2 ± 10.6	33.8 ± 10.7	30.3 ± 10.4	<0.001
Age at last follow-up, y	40.5 ± 10.8	43.0 ± 10.8	39.6 ± 10.7	<0.001
Follow-up duration, y	9.3 (5.0-13.7)	8.7 (4.8-13.6)	9.0 (5.1-13.8)	0.55
Location of referral center (US), %^Ω^	56%	74%	51%	<0.001
Male	58%	58%	59%	0.69
Weight, kg	76 ± 21	77 ± 22	76 ± 20	0.54
BSA	1.9 ± 0.2	1.9 ± 0.3	1.9 ± 0.2	0.71
O_2_ saturations, %	97 ± 3	97 ± 4	97 ± 3	0.87
Morphology – TGA-AS	68%	65%	69%	0.15
VSD	25%	28%	22%	0.04
PA/PS	11%	17%	9%	<0.001
Previous cardiac surgery	26%	34%	23%	<0.001
Diabetes	3%	5%	2%	0.003
Hypertension	8%	9%	7%	0.32
Atrial fibrillation/flutter	27%	39%	23%	<0.001
Ventricular tachycardia	6%	14%	4%	<0.001
PPM	26%	31%	24%	0.005
ICD	4%	7%	3%	<0.001
Medications				
Aspirin	15%	23%	12%	<0.001
Anticoagulation	11%	15%	9%	<0.001
Beta-blocker	21%	29%	18%	<0.001
ACE inhibitor/ARB	35%	42%	33%	0.002
Diuretic agents	12%	23%	9%	<0.001
Spironolactone	5%	10%	4%	<0.001
Pulmonary vasodilators	1%	3%	1%	0.01
Bloodwork	*N = 829*	*N = 290*	*N = 539*	
Hemoglobin, g/dL	14.6 ± 1.9	14.5 ± 1.8	14.6 ± 2.0	0.98
Creatinine, mg/dL	0.9 ± 0.2	0.9 ± 0.2	0.9 ± 0.2	0.51
Albumin, g/dL	4.1 ± 0.5	4.0 ± 0.5	4.3 ± 0.6	<0.001
ECG	*N = 1,548*	*N = 386*	*N = 1,162*	
QRS duration, ms	*115* ± 29	121 ± 31	112 ± 28	<0.001
CPET	*N = 736*	*N = 200*	*N = 536*	
VO_2_ max, mL/kg/min	25.1 ± 9.1	21.5 ± 7.7	26.4 ± 9.3	<0.001
VE/VCO_2_ slope	31 ± 9	32 ± 12	30 ± 6	0.004
Peak heart rate, bpm	153 ± 28	146 ± 28	156 ± 28	<0.001
Transthoracic echocardiogram	*N = 1,628*	*N = 395*	*N = 1,233*	
Right ventricle systolic function				<0.001
Normal	15%	10%	16%	
Mild dysfunction	33%	24%	36%	
Moderate dysfunction	37%	40%	36%	
Severe dysfunction	15%	26%	12%	
Tricuspid regurgitation severity				<0.001
Nil	17%	18%	17%	
Mild	50%	43%	53%	
Moderate	26%	28%	25%	
Severe	7%	11%	5%	
MRI	*N = 611*	*N = 158*	*N = 453*	
RVEF (%)	47 ± 11	44 ± 11	48 ± 11	0.008
LVEF (%)	59 ± 10	58 ± 11	60 ± 10	0.07
RVEDVi (mL/m^2^)	111 ± 39	116 ± 39	109 ± 39	0.05
LVEDVi (mL/m^2^)	76 ± 26	81 ± 30	74 ± 24	0.002
Outcomes				
MCS	1%	4%	0.3%	<0.001
Heart transplant listing	3%	10%	1%	<0.001
Heart transplant	2%	7%	0.5%	<0.001
All-cause mortality	7%	14%	5%	<0.001
All-cause death/heart transplant/MCS	10%	20%	6%	<0.001

Ω Other referral centers are located in Canada, Europe, and New Zealand.

ACE = angiotensin-converting enzyme inhibitors; ARBs = angiotensin receptor blockers BSA = body surface area; CPET = cardiopulmonary exercise test; ECG = electrocardiogram; ICD = implantable cardioverter defibrillator; LVEDVi = left ventricular indexed end diastolic volume; LVEF = left ventricular ejection fraction; MCS = mechanical circulatory support; MRI = magnetic resonance imaging; PA/PS = pulmonary atresia/stenosis; PPM = pacemaker; RVEDVi = right ventricular indexed end diastolic volume; RVEF = right ventricular ejection fraction; TGA-AS = transposition of great arteries palliated with atrial switch repair; VO_2_ = oxygen consumption; VE/VCO2 = ventilatory efficiency; VSD = ventricular septal defect.

Demographic and clinical information for the patients who had catheterization and included in this analysis are presented in Table 2. The mean age at catheterization was 38 ± 11 years. Patients with CCTGA, compared to TGA-AS, were: older at time of catheterization (40 ± 15 vs 37 ± 8 years, *P* = 0.03); more likely to be on diuretic agents; less likely to have a diagnosis of atrial fibrillation; on average have a longer QRS duration; and more likely to have ≥ moderate TR.

**Table 2:** Baseline Characteristics

	Total (N = 261)	TGA-AS (n = 161)	CCTGA (n = 100)	*P* Value^a^
Age at first visit, y	34 ± 11	32 ± 7	39 ± 14	<0.001
Age at time of catheterization, y	38 ± 11	37 ± 8	40 ± 15	0.03
Age at last follow-up, y	44 ± 11	42 ± 7	48 ± 14	<0.001
Follow-up duration postcatheter, y	5.6 (4.8-6.8)	5.2 (3.8-6.0)	7.4 (5.2-9.7)	0.03
VSD	34%	22%	53%	<0.001
PA/PS	21%	11%	38%	<0.001
Previous cardiac surgery^b^	36%	26%	54%	<0.001
PPM	29%	30%	27%	0.60
ICD	7%	8%	7%	0.89
Transthoracic echocardiogram	(n = 252)	(n = 156)	(n = 96)	
Right ventricle systolic function				
Normal	19%	17%	22%	0.43
Mild dysfunction	26%	27%	24%	
Moderate dysfunction	38%	36%	41%	
Severe dysfunction	17%	20%	13%	
Tricuspid regurgitation severity				
Nil	18%	20%	15%	0.005
Mild	40%	45%	30%	
Moderate	30%	27%	35%	
Severe	12%	8%	20%	
MRI	(n = 128)	(n = 80)	(n = 48)	
RVEF	45 ± 8	43 ± 10	49 ± 11	0.002
LVEF	58 ± 9	59 ± 12	55 ± 13	0.07
Hemodynamic parameters				
Cardiac output, L/min	5.1 ± 1.8	5.1 ± 1.6	5.2 ± 2.0	0.71
Cardiac index, L/min	2.7 ± 0.9	2.7 ± 0.8	2.8 ± 1.1	0.43
SVI, mL/m^2^	38 ± 16	38 ± 14	39 ± 17	0.9
API	3.8 ± 2.7	3.9 ± 2.7	3.7 ± 2.5	0.59
PA systolic pressure, mm Hg	41 ± 23	41 ± 25	41 ± 20	0.83
PA diastolic pressure, mm Hg	26 ± 15	27 ± 16	26 ± 14	0.58
Mean PAP, mm Hg mPAP <20 mm Hg mPAP 20-40 mm Hg mPAP 40-60 mm Hg mPAP >60 mm Hg	31 ± 18 34% 43% 16% 7%	32 ± 19 35% 39% 17% 8%	31 ± 16 31% 49% 16% 6%	0.68 0.46
Mean PCWP, mm Hg	16 ± 8	16 ± 8	16 ± 7	0.86
PCWP >15 mm Hg	47%	50%	42%	0.19
RVEDP, mm Hg (n *= 190)*	13 ± 6	12 ± 6	14 ± 7	0.01
Mean CVP, mm Hg	9 ± 5	10 ± 5	9 ± 4	0.63
RA >10 mm Hg	38%	37%	40%	0.66
RA/PCWP	0.6 ± 0.3	0.6 ± 0.3	0.7 ± 0.4	0.64
TPG, mm Hg	15 ± 13	15 ± 15	14 ± 11	0.62
Diastolic pressure gradient, mm Hg	10.2 ± 11.4	10.5 ± 12.4	9.6 ± 9.4	0.53
PVR, WU	3.3 ± 3.6	3.4 ± 3.9	3.3 ± 3.2	0.86
PVR >2 WU	54%	55%	52%	0.68
PVR >5 WU	15%	14%	17%	0.46
PVRi, WU.m^2^	6.2 ± 6.6	6.2 ± 7.0	6.1 ± 6.6	0.89
PVRi >7 WU.m^2^	25%	23%	29%	0.31
PAPI	1.8 ± 1.5	1.8 ± 1.4	1.9 ± 1.8	0.48
PAPI <2	66%	67%	65%	0.73
PAC, mL/mm Hg	6.9 ± 4.9	6.9 ± 4.4	6.9 ± 5.7	0.94
LVSWI, L x mm Hg/m^2^	11 ± 10	12 ± 10	11 ± 10	0.53
RVSWI, L x mm Hg/m^2^	37 ± 16	37 ± 15	37 ± 17	0.88
Pulmonary hypertension type				
Precapillary	24%	22%	27%	0.5
CpcPH	51%	53%	46%	
IpcPH	25%	25%	27%	
Outcomes^c^				
MCS	96% (93-99)	96% (93-100)	96% (91-100)	0.32
Heart transplant	90% (86-94)	88% (82-94)	93% (87-99)	0.89
All-cause mortality	85% (80-91)	86% (79-92)	85% (77-94)	0.78
All-cause death/heart transplant/MCS	75% (69-81)	75% (68-83)	75% (66-85)	0.89

API = aortic pulsatility index; CCTGA = congenitally corrected transposition of the great arteries; CpcPH = combined pre and post capillary pulmonary hypertension; CVP = central venous pressure; IpcPH = isolated post-capillary pulmonary hypertension; LVSWI = LV stroke work index; mPAP = mean pulmonary artery pressure; PA = pulmonary artery; PAC = pulmonary artery compliance; PAP = pulmonary artery pressure; PAPI = pulmonary artery pulsatility index; PCWP = pulmonary artery wedge pressure; PCWP = pulmonary capillary wedge pressure; PVR = pulmonary vascular resistance; RA = right atrium; RVSWI = RV stroke work index; RVEDP = Right ventricular end diastolic pressure; SVI = indexed stroke volume; TPG = transpulmonary gradient; WU = Wood units; other abbreviations as in Table 1.

a Other than atrial switch operation, including: tricuspid valve surgery 5% (1% TGA-AS vs 12% CCTGA, *P* < 0.001), VSD closure 19% (7% vs 39%, *P* < 0.001), LV outflow tract surgery 15% (3% vs 33%, *P* < 0.001), other 22% (palliative shunt 4% (n = 10), PA banding 4% (n = 11), coarctation surgery 2% [n = 4]).

b Comparisons analyzed with univariate Cox regression.

c Estimated survival from outcome at 5 years post catheterization.

Associated cardiac diagnoses included: VSD 34% (22% TGA-AS vs 53% CCTGA; *P* < 0.001), pulmonary atresia or stenosis 21% (11 v 38%; *P* < 0.001), situs inversus 4% (0 v 10%; *P* < 0.001), heterotaxy 1% (0.6 v 2%; *P* = 0.31), and criss-cross heart 1% (0.6 v 2%; *P* = 0.31). Overall, 36% had undergone cardiac surgery (other than atrial switch).

### Pulmonary hypertension subtypes

A diagnosis of PH (mean PAP > 20 mm Hg) was present in 66% of patients (TGA-AS in 65% v CCTGA 69%; *P* = 0.74). If a threshold of mPAP >25 mm Hg was used, PH was present in 54% (52% vs 56%; *P* = 0.4)

PH, when present, was classified as precapillary in 24%, CpcPH in 51%, and IpcPH in 25% (Central Illustration). Using the 2015 ESC classification, a greater proportion would have been classified as IpcPH rather than CpcPH (precapillary 25%, CpcPH 35%, and IpcPH 39%). There was no significant difference in PH subtype between TGA-AS and CCTGA, whether using the 2015 or 2022 ESC definition.

**Central Illustration:**
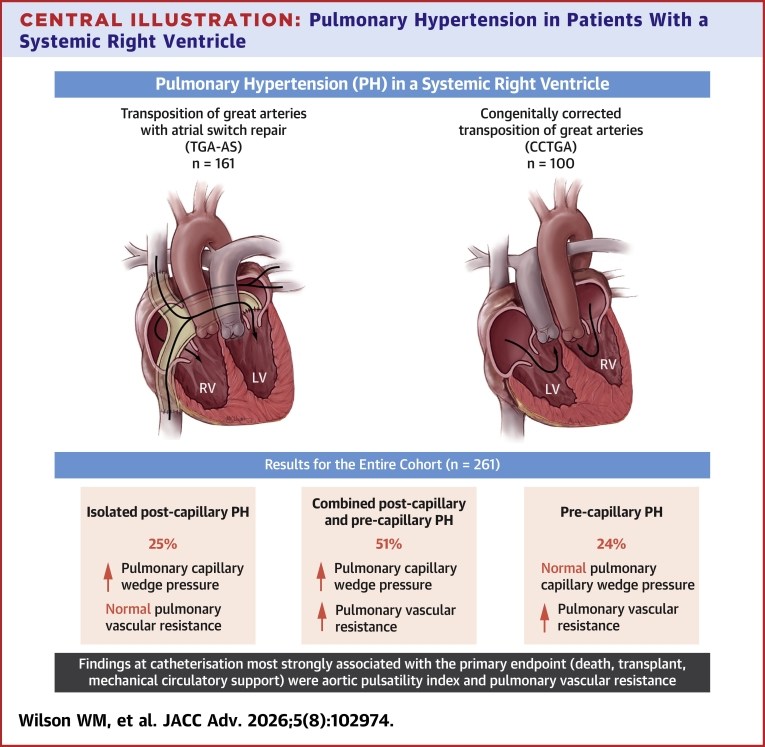
**Pulmonary Hypertension in Patients With a Systemic Right Ventricle** Pulmonary hypertension is common in patients with a systemic right ventricle undergoing catheterization and is strongly associated with adverse clinical outcomes. The majority of patients with pulmonary hypertension in this setting have a combined pre and post capillary subtype with a combination of elevated filling pressures and elevated pulmonary vascular resistance. Pulmonary vascular resistance and aortic pulsatility index are predictors of adverse events.

**Figure 2:**
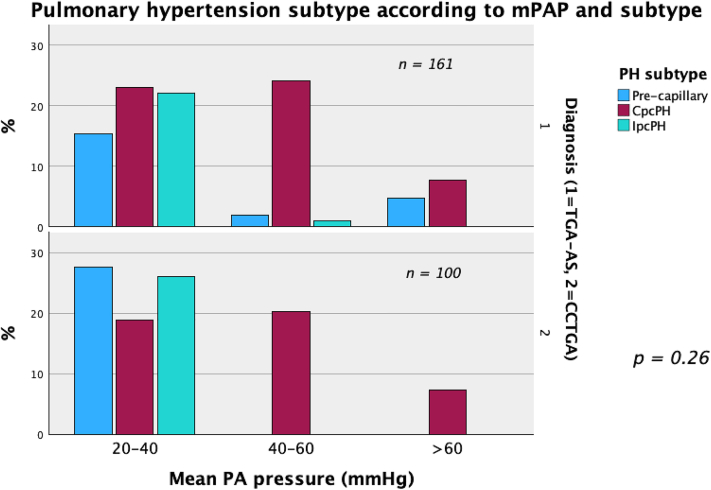
**Pulmonary Hypertension Subtype According to Diagnosis and PH Severity** Chi-square test was used to assess whether frequencies of patients classed by PH type and mean PAP differed by TGA morphology. IpcPH = isolated post capillary pulmonary hypertension; CpcPH = combined precapillary and postcapillary pulmonary hypertension; mPAP = mean pulmonary artery pressure; PA = pulmonary artery; PH = pulmonary hypertension; TGA-AS = transposition of great arteries palliated with atrial switch repair.

**Figure 3:**
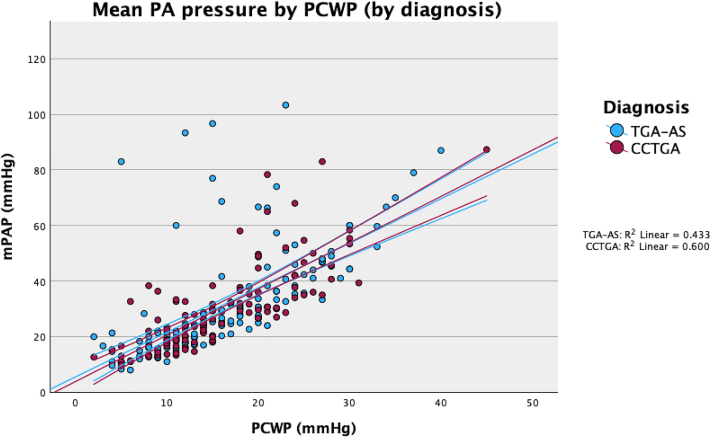
**Scatterplot of mPAP vs PCWP by Diagnosis (Line of Best Fit and 95% Error Margins)** CCTGA = congenitally corrected transposition of the great arteries; PCWP = pulmonary capillary wedge pressure; other abbreviations as in Figure 2.

Subtype classification according to diagnosis and PH severity is presented in Figure 2 and relationship of mean PAP to PCWP stratified by diagnosis presented in (Figure 3, Supplemental Figure 1A). The relationship of mPAP to PCWP is presented in Supplemental Table 3.

Overall, the relationship between PCWP and mPAP was similar between the 2 groups; nearly all with a mPAP >40 mm Hg had a PCWP >15 mm Hg (88% in TGA-AS subgroup and 95% in CCTGA subgroup). In the TGA-AS subgroup, greater variability in PCWP was evident when mPAP was significantly elevated with a proportion of patients having normal PCWP (ie precapillary subtype). For example, if mPAP is >40 mm Hg, there is a greater proportion of the precapillary subtype in TGA-AS compared to CCTGA (17% vs 5%; *P* < 0.05). In addition, if mean PAP is >60 mm Hg, only 69% of patients with TGA-AS had a PCWP >15 mm Hg compared to 100% with CCTGA (*P* < 0.05).

Most patients with PH had an elevated PVR, in particular if mPAP >40 mm Hg (Supplemental Figure 1B). The majority of patients with PH had a PVR >2 WU and if mean PAP was more than 60 mm Hg, nearly all had a PVR >5 WU.

### Factors associated with PH and elevated PVR

Differences in baseline variables between those with PH vs not and those with elevated PVR vs not are presented in Supplemental Tables 4 and 5. Those with PH were older (44 ± 11 vs 40 ± 9 years; *P* = 0.01). Patients with TGA-AS who had a VSD were more likely to have PH (28% vs 11%; *P* = 0.03) and an elevated PVR (30% vs 14%;*P* = 0.02). Furthermore, TGA-AS patients with precapillary PH were more likely to have had a VSD (44% precapillary vs 28% CpcPH vs 17% IpcPH; *P* = 0.04). Age at time of atrial switch operation was 2.4 ± 2.0 years in those with a PVR >2 WU vs 1.9 ± 1.7 years if not (*P* = 0.12). In those with TGA-AS, those with IpcPH were more likely to be male (87% IpcPH vs 57% precapillary and 61% CpcPH; *P* = 0.04).

PH was present in 32% of patients with CCTGA who had pulmonary stenosis compared to 52% in those who did not (*P* = 0.08).

### Factors associated with adverse clinical events

Hemodynamic parameters stratified according to diagnosis and occurrence of clinical event are presented in Table 3. There was no significant difference in the proportion of PH subtypes in those who had an event (precapillary 18%, CpcPH 63%, and IpcPH 19%) and did not (precapillary 27%, CpcPH 46%, IpcH 27%; *P* = 0.10). CpcPH was more common in TGA-AS patients who had an event compared to those who did not (*P* = 0.04).

**Table 3:** Hemodynamic Parameters and Clinical Outcomes

	TGA-AS			CCTGA		
	Event (n = 39)	No Event (n = 122)	*P* Value	Event (n = 29)	No Event (n = 71)	*P* Value
Cardiac output, L/min	4.9 ± 1.6	5.2 ± 1.6	0.36	4.7 ± 1.4	5.4 ± 2.1	0.10
Cardiac index, L/min/m^2^	2.6 ± 0.7	2.7 ± 0.9	0.59	2.5 ± 0.7	3.0 ± 1.2	0.04
SVI, mL/m^2^	37.3 ± 12.6	38.5 ± 14.3	0.66	34 ± 12	41 ± 18	0.048
API	2.7 ± 2	4.2 ± 2.9	0.003	2.5 ± 1.4	4.2 ± 2.8	0.002
PASP, mm Hg	59 ± 30	36 ± 19	<0.001	51 ± 21	36 ± 19	0.001
PASP/SBP	0.51 ± 0.26	0.30 ± 0.17	<0.001	0.5 ± 0.2	0.3 ± 0.2	0.001
PA diastolic pressure, mm Hg	38 ± 19	23 ± 13	<0.001	32 ± 15	23 ± 12	0.003
Mean PAP, mm Hg	45 ± 23	27 ± 15	<0.001	38 ± 17	27 ± 14	0.002
>20	85%	62%	0.01	90%	61%	0.004
>40	51%	17%	<0.001	41%	11%	<0.001
>60	23%	3%	<0.001	7%	4%	0.58
Mean PCWP, mm Hg	21 ± 9	15 ± 7	<0.001	21 ± 8	14 ± 6	<0.001
PCWP >15	74%	43%	<0.001	66%	32%	0.002
PCWP >20	49%	22%	0.001	55%	14%	<0.001
PCWP >25	31%	7%	<0.001	28%	4%	<0.001
DPG, mm Hg	17.5 ± 17.5	8.3 ± 9.5	<0.001	11.5 ± 10.6	8.9 ± 8.8	0.004
DPG >7 mm Hg	67%	41%	0.005	48%	47%	0.87
Mean RA, mm Hg	12 ± 6	9 ± 4	0.003	10 ± 4	9 ± 4	0.63
RA >10 mm Hg	62%	38%	<0.001	59%	61%	0.86
RAP/PCWP	0.6 ± 0.3	0.7 ± 0.3	0.26	0.5 ± 0.3	0.7 ± 0.4	0.004
TPG, mm Hg	24 ± 21	12 ± 11	<0.001	17 ± 11	13 ± 10	0.09
PVR, WU	5.5 ± 5	2.6 ± 3.2	<0.001	4.1 ± 3.6	2.9 ± 3.0	0.09
PVR >2	77%	48%	0.001	72%	44%	0.009
PVR >5	39%	6%	<0.001	31%	11%	0.02
PAPI	2.2 ± 1.7	1.6 ± 1.2	0.03	2.1 ± 1.2	1.8 ± 1.9	0.38
PAPI <2	56%	71%	0.10	62%	66%	0.70
LVSWI (subpulmonic), L x mm Hg/m^2^	17.6 ± 13.8	9.8 ± 8.1	<0.001	13 ± 8	10 ± 10	0.18
RVSWI (systemic), L x mm Hg/m^2^	37.0 ± 13.1	40.2 ± 16.4	0.28	32.2 ± 11.2	40.3 ± 17.4	0.02
PAC, mL/mm Hg	5.3 ± 4.9	7.5 ± 4.1	0.007	4.3 ± 2.5	7.9 ± 6.3	0.004
Cardiac power	0.9 ± 0.3	1.0 ± 0.4	0.29	0.9 ± 0.2	1.0 ± 0.4	0.12
CPI	0.5 ± 0.2	0.5 ± 0.2	0.49	0.5 ± 0.1	0.6 ± 0.2	0.05
PCWP/CI	8.4 ± 4.4	5.9 ± 3.4	<0.001	9.4 ± 6	5.8 ± 3.8	<0.001
PH subtype						
Precapillary	12%	27%	0.04	27%	26%	0.8
CpcPH	73%	47%		50%	44%	
IpcPH	15%	27%		23%	30%	

CI = cardiac index, CPI = cardiac power index; DPG = diastolic pressure gradient; PASP = pulmonary artery systolic pressure; RAP = right atrial pressure; SBP = systolic blood pressure; other abbreviations as in Tables 1 and 2.

Univariable analysis of hemodynamic parameters associated with the primary endpoint are presented in Table 4.

**Table 4:** HRs From Univariable Cox Proportional Hazards Models for Freedom From MACE

	Overall	TGA-AS	CCTGA
Age, 30-45 y (vs 0-30)	3.0 (1.4-6.4), *P* = 0.006	3.3 (1.1-9.4), *P* = 0.03	2.3 (0.6-8.8), *P* = 0.02
Age, 45-60 y (vs 0-30)	5.6 (2.4-13.1), *P* < 0.001	5.3 (1.4-20.0), *P* = 0.01	6.6 (1.9-23.4), *P* = 0.003
Age >60 y (vs 0-30)	5.0 (1.7-14.5), *P* = 0.003	NA	5.6 (1.4-22.5), *P* = 0.015
mPAP >20	4.2 (2.1-8.4), *P* < 0.001	3.2 (1.4-7.7), *P* = 0.008	6.4 (1.9-21.1), *P* = 0.003
mPAP >40	3.5 (2.2-5.7), *P* < 0.001	3.6 (1.9-6.8), *P* < 0.001	3.6 (1.7-7.5), *P* < 0.001
mPAP >60	3.4 (1.8-6.6), *P* < 0.001	4.5 (2.1-9.5), *P* < 0.001	1.8 (0.4-7.6), *P* = 0.40
PASP/SBP >0.3	3.4 (2.0-5.9), *P* < 0.001	2.9 (1.5-5.7), *P* = 0.002	5.0 (2.0-12.4), *P* < 0.001
PCWP >15	3.2 (1.9-5.5), *P* < 0.001	3.10 (1.5-6.5), *P* = 0.002	3.4 (1.6-7.4), *P* = 0.002
PCWP >20	3.5 (2.1-5.6), *P* < 0.001	3.10 (1.5-6.5), *P* = 0.002	4.9 (2.3-10.2), *P* < 0.001
PCWP >25	4.4 (2.6-7.5), *P* < 0.001	4.40 (2.2-8.7), *P* < 0.001	4.5 (2.0-10.2), *P* < 0.001
DPG >7	1.90 (1.2-3.0), *P* = 0.01	2.70 (1.4-5.4), *P* = 0.004	1.30 (0.6-2.8), *P* = 0.40
TPG >15	2.80 (1.7-4.5), *P* < 0.001	3.10 (1.6-5.8), *P* < 0.001	2.70 (1.3-5.7), *P* = 0.007
PVR >2	3.20 (1.8-5.5), *P* < 0.001	3.00 (1.4-6.3), *P* = 0.004	3.70 (1.6-8.4), *P* = 0.002
PVR >5	4.30 (2.6-7.1), *P* < 0.001	5.10 (2.7-9.8), *P* < 0.001	3.70 (1.7-8.1), *P* = 0.001
PVRi >7	3.40 (2.1-5.4), *P* < 0.001	3.90 (2.1-7.4), *P* < 0.001	3.50 (1.7-7.3), *P* < 0.001
API <1.5	5.10 (2.9-8.7), *P* < 0.001	7.40 (3.4-16.1), *P* < 0.001	4.20 (1.9-9.3), *P* < 0.001
API <3	2.90 (1.7-4.8), *P* < 0.001	2.70 (1.4-5.4), *P* = 0.004	3.20 (1.5-7.0), *P* = 0.003
PAC <6	3.00 (1.7-5.3), *P* < 0.001	2.60 (1.2-5.6), *P* = 0.01	3.80 (1.6-8.9), *P* = 0.002
LVSWI >10	2.70 (1.7-4.5), *P* < 0.001	2.70 (1.7-4.5), *P* < 0.001	2.60 (1.2-5.4), *P* = 0.01
RVSWI <35	1.90 (1.2-3.2), *P* = 0.009	1.60 (0.8-3.1), *P* = 0.10	2.40 (1.1-5.3), *P* = 0.02
CI <2.5	1.25 (0.8-2.0), *P* = 0.40	0.91 (0.5-1.7), *P* = 0.9	1.9 (0.9-3.3), *P* = 0.09
PCWP/CI > 5	3.10 (1.8-5.4), *P* < 0.001	2.60 (1.2-5.6), *P* = 0.01	3.80 (1.6-8.9), *P* = 0.002
SVI <35	1.4 (0.8-2.5), *P* = 0.20	0.91 (0.5-1.7), *P* = 0.90	2.4 (1.1-5.0), *P* = 0.03
RA >10	2.20 (1.3-3.5), *P* = 0.002	3.30 (1.7-6.4), *P* < 0.001	1.10 (0.5-2.2), *P* = 0.90
RA/PCWP <0.6	1.52 (0.9-2.5), *P* = 0.09	1.01 (0.5-1.9), *P* = 1.00	3.0 (1.4-10), *P* = 0.008
PAPI>2	1.40 (0.8-2.2), *P* = 0.20	1.50 (0.8-2.8), *P* = 0.20	1.20 (0.6-2.6), *P* = 0.60
CPI <0.5	1.50 (0.9-2.4), *P* = 0.10	1.10 (0.6-2.0), *P* = 0.80	2.40 (1.1-5.2), *P* = 0.02

Terms shown in the same cell represent the different univariable Cox models but with different thresholds for the predictor (eg, for mPAP three different models were fitted, using three thresholds for this variable); the exception here was age at catheterization, in which case a single model was fitted with a single ordinal categorical predictor, and the HRs are with respect to the reference class (0-30 years). Note that there were no TGA-AS patients aged >60 in the cohort.

Abbreviations as in Tables 1 to 3.

**Figure 4:**
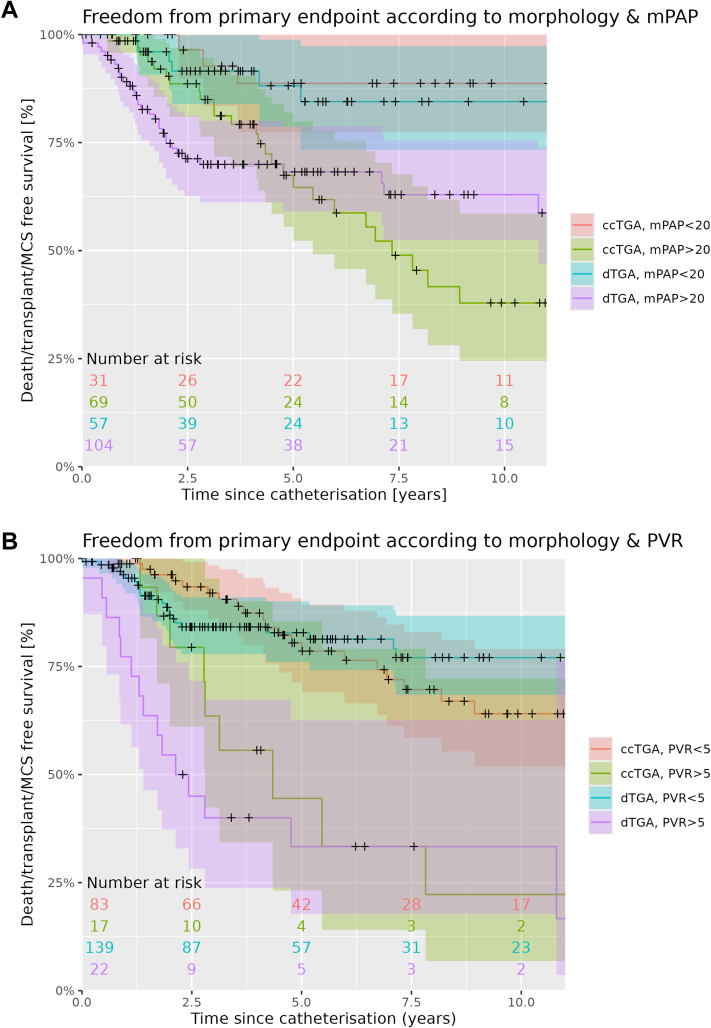
Freedom From Primary Endpoint According to Morphology and PH/PVR (A) Freedom from primary endpoint according to diagnosis and pulmonary HT. (B). Freedom from primary endpoint according to diagnosis and PVR. MCS = mechanical circulatory support; PVR = pulmonary vascular resistance; TGA = transposition of great arteries; other abbreviations as in Figures 2 and 3.

Freedom from the primary endpoint according to mPAP and PVR stratified by diagnosis is presented in Figure 4.

In both CCTGA and TGA-AS, the primary endpoint was associated with a lower API, higher PAPs, higher PCWP, higher PCWP/CI ratio, and lower PAC. There were differences in predictors of the primary endpoint between diagnoses on univariable analysis: in CCTGA patients (but not TGA-AS patients), the primary endpoint was associated with a lower cardiac power index, lower indexed stroke volume, lower RA/PCWP ratio and lower RVSWI; in TGA-AS, the primary endpoint was associated with a higher PVR, higher TPG, higher PAPI and higher RA pressure.

**Figure 5:**
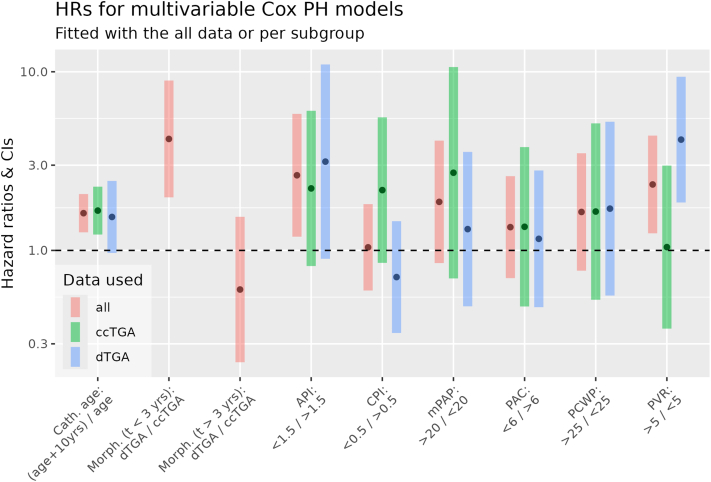
**Multivariable Analysis for Predictors of Primary Outcome (HR for Death/Transplant/MCS)** API = aortic pulsatility index; CPI = cardiac power index; PAC = pulmonary artery compliance; other abbreviations as in Figures 2, 3, and 4.

A multivariable model was created to evaluate factors associated with the primary endpoint (C-statistic = 0.79; SE = 0.03); findings stratified by underlying diagnosis are presented in (Figure 5, Table 5). The primary endpoint was associated with age, API <1.5, PVR >5 WU, TGA-AS in the period 0 to 3 years postcatheterization, and CCTGA for >3 years postcatheterization. The predictive power of PVR appeared to be confined to the TGA-AS subgroup.

**Table 5:** HRs and the Corresponding 95% CIs for Multivariable Cox Regression Models for Prediction of the Primary Endpoint

	Overall (Full Cohort) HR (95% CI)	TGA-AS Data Only HR (95% CI)	CCTGA Data Only HR (95% CI)
Age, decades	1.6 (1.2-2.1), *P* < 0.001	1.5 (0.97-2.5), *P* = 0.068	1.7 (1.3-2.2), *P* = 0.001
API <1.5	2.6 (1.2-5.8), *P* = 0.02	3.1 (0.9-11.0), *P* = 0.07	2.2 (0.82-6.0), *P* = 0.12
PVR >5	2.3 (1.2-4.3), *P* = 0.008	4.2 (1.8-9.4), *P* < 0.001	1.0 (0.4-2.9), *P* = 0.94
PCWP >25	1.6 (0.8-3.5), *P* = 0.19	1.7 (0.6-5.2), *P* = 0.34	1.7 (0.5-5.1), *P* = 0.38
mPAP >20	1.9 (0.9-4.2), *P* = 0.12	1.3 (0.5-3.6), *P* = 0.58	2.7 (0.7-10.6), *P* = 0.14
CPI <0.5	1.04 (0.5-1.8), *P* = 0.88	0.8 (0.3-1.5), *P* = 0.34	2.2 (0.9-5.6), *P* = 0.10
PAC <6	1.4 (0.7-2.6), *P* = 0.37	1.2 (0.5-2.8), *P* = 0.74	1.4 (0.5-3.8), *P* = 0.56
Morphology (TGA-AS)			
<3 y	4.2 (2.0-8.9), *P* < 0.001		
>3 y	0.6 (0.2-1.5), *P* = 0.29		

Each column provides results for separate Cox models.

Abbreviations as in Tables 1 to 3.

## Discussion

In this large hemodynamic study of patients with a two-ventricle systemic RV (2V-RV), we demonstrate the PH is prevalent among those referred for catheterization. The distribution of PH subtypes was broadly similar in both subgroups (TGA-AS and CCTGA), with combined precapillary and postcapillary PH predominating.

Despite the overall similarity, some important differences were observed between underlying diagnoses. A subset of TGA-AS patients had precapillary PH, particularly at higher mPAP thresholds, consistent with severe pulmonary vascular disease. By contrast, PH in CCTGA was almost exclusively postcapillary at higher pulmonary pressures. Clinical correlates also differed between groups; in TGA-AS, a history of VSD was associated with PH and elevated PVR, whereas in CCTGA, pulmonary stenosis appeared protective. Factors associated with adverse clinical outcomes were lower API and elevated PVR; there was a stronger association between PVR elevation and the primary endpoint, in particular, in the TGA-AS subgroup compared to CCTGA, potentially indicative of differing underlying pathophysiological drivers.

This study provides several important advances over previous research. It represents the largest hemodynamic phenotyping study of a 2V-RV population to date, allowing more robust characterization of PH prevalence and subtype distribution across both TGA-AS and CCTGA. This study compares findings between TGA-AS and CCTGA, providing novel insights into condition-specific mechanisms and risk stratification. Furthermore, this is the first study to apply the contemporary definition of PH.

The prevalence of PH complicating TGA-AS and CCTGA is not well defined. This partially relates to the challenges in diagnosis of PH noninvasively in 2V-RV patients, in particular via echocardiography where left ventricular systolic pressure is difficult to define owing to lack of mitral regurgitation. Systematic invasive catheterization, ideally performed by operators with ACHD expertise, in all 2V-RV patients aged ≥40 years has been advocated by some for this reason.^2^ PH, in the absence of anatomical causes such as pulmonary venous baffle obstruction, has been reported in 7% to 8% of all-comers with TGA-AS,^14^, ^15^, ^16^ whilst the prevalence in those who have undergone catheterization ranges between 48% to 54% in small single-center studies using mPAP >25 mm Hg as the threshold.^2^^,^^6^^,^^9^ The higher prevalence of PH in our study likely reflects use of contemporary criteria (66% if PH defined as mPAP >20 mm Hg, 54% if defined as mPAP >25 mm Hg). Heart failure has been reported as a common late issue in CCTGA but there are few reports regarding pulmonary hypertension as a complication of this condition.^2^^,^^17^

There is conjecture whether the primary mechanism for PH in TGA-AS patients relates to abnormal pulmonary vasculature (ie precapillary pulmonary HT)^7^^,^^15^^,^^18^ or elevated filling pressures (ie post capillary pulmonary HT, which may relate to RV systolic or diastolic dysfunction, TR, poor atrial transport, and pulmonary venous baffle stenosis.).^2^^,^^6^ All patients in small catheterization based studies by Chaix et al^6^ (Montreal) and Miranda et al^9^ (Mayo) had a postcapillary subtype (IpcPH or CpcPH) with elevation of PCWP. In the Mayo study, there was a higher proportion of PCWP elevation (59%) compared to right ventricular end diastolic pressure (RVEDO) elevation (35%) suggesting decreased pulmonary baffle compliance and/or pulmonary venous baffle obstruction was contributory to pulmonary venous hypertension in some. In a study by Van de Bruaene et al (Toronto), approximately 80% of the TGA-AS patients (n = 48) with PH had postcapillary PH (precapillary 12%, CpcPH 42%, IpcPH 40% and pulmonary baffle stenosis in 6%). Our findings suggest a similar spectrum of disease; whilst the majority have CpcPH, a subset demonstrate severe precapillary PH; if mPAP was more than 40 mm Hg, the majority of TGA-AS patients were CpcPH but ∼20% were precapillary (compared to CCTGA where almost all were CpcPH subtype). Precapillary PH in TGA-AS patients may relate to: abnormal fetal circulation (increased pulmonary blood flow in the fetus and distribution of oxygenated blood through the fetal vascular bed;^8^ presence of a shunt (VSD or patent ductus arteriosus); timing of repair.^12^^,^^19^ In our study, age at atrial switch was numerically higher in precapillary or CpcPH subtypes; however, overall there was no significant difference between subtypes.

The mechanism for PH in CCTGA is not well described but the majority of those with PH in a single-center Toronto study referenced previously were postcapillary in nature (precapillary 7%, CpcPH 54%, and IpcPH 39%).^2^ Overall, our findings suggest a similar predisposition to pulmonary HT between the 2 diagnoses, which presumably relates, in part, to a maladapted subaortic RV and tricuspid valve prone to failure. The majority of patients with a mPAP>40 mm Hg also have elevation in PVR, suggestive of coexistent pulmonary vascular disease, a pattern which appears more pronounced in the TGA-AS group.

PH in our study was associated with adverse clinical outcomes in keeping with previous reports in patients with a systemic RV.^2^^,^^6^^,^^9^ In a single-center study by Van de Bruaene et al, BNP elevation provided additional prognostic value but there was no difference in clinical events according to PH subtype. The combined (CpcPH) subtype was associated with a higher major adverse cardiovascular events rate in our study and PVR elevation was a strong predictor of adverse clinical events, particularly in the TGA-AS group. Using this data set (excluding those with severe PH), we have previously reported API to be a strong predictor of clinical events in patients with a systemic RV;^20^ further analysis in this paper suggests a low API, characterized by a low pulse pressure and high PCWP reflective of decreased myocardial efficiency, is a predictor of adverse events in both TGA-AS and CCTGA patients.

Characterization of the mechanism for PH has implications for management of patients with heart failure and a systemic RV. The findings that PH is largely driven by pulmonary venous hypertension assumes greater importance as these patients develop advanced heart failure when co-existent PH may be considered a contraindication to heart-only transplantation. Diuresis, inotrope, or MCS use may reduce PAP significantly by lowering filling pressures, rendering one suitable for heart-only rather than heart-lung transplantation.^2^ Repeat catheterization after IV diuresis to reassess hemodynamics in a “dry” state is of value in this setting, whilst ventricular assist device as a bridge to transplant should be considered if medical therapy is ineffective in reducing PA pressures as prolonged subaortic ventricular unloading can reverse PH with reduction PA pressures as well as TPG and PVR.^2^^,^^21^^,^^22^ Pulmonary vasodilators may be harmful in this context; however, a subset of patients, in particular those with TGA-AS, appear prone to severe precapillary PH, in which case pulmonary vasodilator use could be considered although there are mixed reports regarding their benefit in this context.^7^^,^^18^^,^^23^, ^24^, ^25^

### Study Limitations

This retrospective analysis of a referral population is subject to limitations, in particular, nonstandardized indications for cardiac catheterization. These findings are, therefore, not applicable to all patients with a systemic RV. It is also worth acknowledging that any differences between TGA-AS and CCTGA patients in this study may reflect the different survival trajectory of the 2 groups (with lower event rate and smaller sample size in the CCTGA group).

Interpretation of PCWP in patients with TGA after atrial switch can be fraught owing to the presence of large V waves, attributable most often to a combination of systemic atrioventricular valve regurgitation and a stiff atrial (baffle) chamber. The available data included only mean PCWP and we do not have information regarding how each laboratory measured PCWP. Furthermore, infrequent estimates of RVEDP were provided, thus we were unable to include a PCWP-RVEDP comparison, which would be of interest in the TGA-AS group, in particular.

A change in definition of PH and PH subtypes affects direct comparison with findings of studies published before 2022.

Comparison between invasive hemodynamics and echocardiography findings, in particular severity of RV dysfunction and TR, would be of interest; echocardiography was not performed contemporaneously with catheterization; therefore, we have not explored this relationship in this manuscript.

Central aortic pressures were not universally recorded; therefore, parameters using aortic blood pressure (ie API, cardiac power, RVSWI) were calculated using noninvasive brachial blood pressure measurements. RVSWI was calculated using PCWP rather than RVEDP, which was not available in all patients and may have affected validity in TGA-AS patients, in particular. Cardiac output calculation in this study was via thermodilution or the indirect Fick method if the thermodilution method was not reported; previous reports have described only modest correlation between thermodilution and Fick cardiac output calculations.^26^

Furthermore, we do not have data regarding the use of sedation or general anesthesia at the time of catheterization, which may have affected measurements.

The three multivariable Cox regression models had relatively low numbers of events per parameter, especially when restricted to the CCTGA or TGA-AS subgroups (7.5 events per parameter for full cohort model, and 4.1 or 5.6 events per parameter for the subgroup analyses); these subgroup analyses are likely more prone to higher type 1 error rates.^27^

## Conclusions

PH is common in patients with a systemic RV undergoing catheterization and is associated with adverse clinical outcomes. A high index of suspicion and a low threshold to perform catheterization is warranted. The majority of patients with PH in this setting have a combination of elevated filling pressures and elevated PVR. Severe precapillary PH occurs in a small proportion of patients with TGA-AS.

Perspectives **COMPETENCY IN MEDICAL KNOWLEDGE:** PH is common in patients with a 2V-RV circulation referred for catheterization; therefore, a low threshold for catheterization is reasonable in those aged more than 40 years. PH is likely multifactorial and the most common subtype is a combined picture (CpcPH). PH is associated with adverse clinical outcomes.

**TRANSLATIONAL OUTLOOK:** In 2V-RV patients with PH, the majority have a postcapillary picture with PCWP elevation. Repeat catheterization after optimization of heart failure therapy and diuresis may be appropriate to reassess hemodynamics in an “optimized” clinical state. In the setting of advanced heart failure with PH, reassessment of hemodynamics after subaortic ventricular unloading (ie inotropes or ventricular assist device) is important to define transplant candidacy. The findings of this study apply to those referred for catheterization, thus cannot necessarily be extended to all 2V-RV patients.

## Funding support and author disclosures

The authors have reported that they have no relationships relevant to the contents of this paper to disclose.

## Supplementary materials

Supplemental_Material_CLEAN
