# Supplementary material for: Pulmonary Hypertension in Adults With a Systemic Right Ventricle (Biventricular Circulation)
Source: JACC Adv. 2026 Jul 2;5(8):102974. doi: 10.1016/j.jacadv.2026.102974 (PMC13351456; doi:10.1016/j.jacadv.2026.102974)

**eSUPPLEMENTAL MATERIAL**

**Supplementary Table S1. Equations employed to compute hemodynamic parameters based on cardiac catheterization data.**

| **Measured parameter** |  |
| --- | --- |
| Mean central venous pressure |  |
| Pulmonary artery systolic pressure |  |
| Pulmonary artery diastolic pressure |  |
| Mean pulmonary artery pressure |  |
| Mean pulmonary capillary wedge pressure |  |
| Non-invasive brachial systolic blood pressure |  |
| Non-invasive brachial diastolic blood pressure |  |
| Mean systemic blood pressure |  |
| Heart rate |  |
| **Calculated parameter** | |
| Aortic pulsatility index | (SBP-DBP)/PCWP |
| Systemic vascular resistance index | (Mean systemic blood pressure – mean central venous pressure)/ cardiac index |
| Left ventricular stroke work index | 0.0136 X [SVI X (mean pulmonary artery blood pressure – LVEDP)] |
| Transpulmonary gradient | Mean pulmonary artery pressure - mean pulmonary capillary wedge pressure |
| Pulmonary vascular resistance index | (Mean pulmonary artery pressure - mean pulmonary capillary wedge pressure)/ cardiac index |
| Right ventricular stroke work index | 0.0136 X [SVI X (mean systemic blood pressure – PCWP) |
| Pulmonary artery compliance | Stroke volume / PA pulse pressure (PAS-PAD) |
| Diastolic pressure gradient | PA diastolic - PCWP |
| Pulmonary artery pulsatility index | (Pulmonary artery systolic pressure - Pulmonary artery diastolic pressure)/ mean central venous pressure |
| Indirect cardiac index using Fick method | Oxygen consumption/[SaO_2-_ SvO_2_] X Hemoglobin X 13.6)/ body surface area |
| Cardiac index using thermodilution | Method determined by rapid-injection 10 mL of iced (4° C) or room temperature (20° C) saline injected as the indicator through the proximal port of the PAC. Complete mixing of the injectate with blood causes a decrease in blood temperature, detected by the distal thermistor. The cardiac index computer calculates the change in indicator concentration (temperature over time) to determine cardiac index |
| Cardiac power | Mean arterial pressure x CO x 0.0022 |

Abbreviations: SBP= Systolic blood pressure, DBP= Diastolic blood pressure, PCWP= Pulmonary capillary wedge pressure, SVI = Stroke Volume Index, LVEDP = Left Ventricle End-Diastolic Pressure, RVEDP = Right Ventricle End-Diastolic Pressure, SaO2 = Systemic Arterial Oxygen Saturation, SvO2 = Mixed Venous Oxygen Saturation

**Supplementary Table S2. Referral source for catheterisation**

Patients referred for catheterisation represented 24% of the overall study group and were more likely to be from the US compared to those who did not have a catheter study (74% versus 51%, p<0.001). The proportions were similar between underlying diagnoses*.*

|  | All | Catheter study | If catheter study: US (%) | No catheter study | If no catheter study: US (%) |
| --- | --- | --- | --- | --- | --- |
| Total | 1721 | 410 (24%) | 303 (74%) | 1311 (76%) | 669 (51%) |
| TGA-AS | 1163 | 270 (23%) | 200 (74%) | 893 (77%) | 491 (55%) |
| CCTGA | 558 | 140 (25%) | 104 (74%) | 418 (75%) | 209 (50%) |

**Supplementary Table S3. PAP severity and relationship to PCWP, PVR**

|  | Total | TGA | CCTGA | p |
| --- | --- | --- | --- | --- |
| **mPAP>20mmHg** | 68% (n=177) | 67% (n=108) | 69% (n=69) | 0.75 |
| PCWP  PCWP>15  PCWP >20 | 19.5±6.8  70%  41% | 19.9±6.8  75% (n=81)  43% | 18.9±6.9  61% (n=42)  38% | 0.56  0.04  0.24 |
| PVR  PVR>2  PVR>5  Subtype (pre/CpcPH/IpcPH) (%) | 4.2±4.1  71%  22%  24/51/25 | 4.2±4.4  71% (77)  19% (21)  22/55/23 | 4.2±3.5  71% (49)  24% (17)  26/46/28 | 1.0  0.73  0.46  0.55 |
| **mPAP >40** | 23% (n=61) | 26% (n=41) | 20% (n=20) | 0.22 |
| PCWP  PCWP>15  PCWP >20 | 24.8±6.9  90% (55)  77% | 24.7±7.2  88% (36)  78% | 25.0±6.3  95% (19)  75% | 0.87  0.19  0.65 |
| PVR  PVR>2  PVR>5  Subtype (pre/CpcPH/IpcPH) (%) | 7.6±5.2  98%  52% (32)  13/85/2 | 7.6±5.2  100%  49% (20)  17/81/2 | 7.8±4.3  98%  60% (12)  5/95/0 | 0.88  0.68  0.46  0.32 |
| **mPAP >60** | 7% (n=18) | 8% (n=13) | 5% (n=5) |  |
| PCWP  PCWP>15  PCWP>20 | 24.1±10.5  78% (14)  72% (13) | 22.7±10.7  69% (9)  54% (7) | 27.6±10.0  100% (5)  100% (5) | 0.37  0.19  0.04 |
| PVR  PVR>2  PVR>5  Subtype (pre/CpcPH/IpcPH) (%) | 12.3±6.4  100% (18)  89% (16)  28/72/0 | 13.1±6.4  100% (13)  92% (12)  39/62/0 | 10.4±6.2  100% (5)  80% (4)  0/100/0 | 0.43  0.68  0.46  0.1 |

Supplementary Table S4. PAP and PVR according to baseline variables

|  | **mPAP>20** | | | **mPAP>40** | | | **PVR>2** | | |
| --- | --- | --- | --- | --- | --- | --- | --- | --- | --- |
|  | **Overall** | **TGA-AS** | **CCTGA** | **Overall** | **TGA** | **CCTGA** | **Overall** | **TGA-AS** | **CCTGA** |
| Age | 44±11 v 40±9, p=0.01 | 42±8v39±6, p=0.01 | 49±14v44±13, p=0.2 | 45±10 v 43±11, p=0.25 | 42±8v41±7, p=0.3 | 50±13v46±14, p=0.3 | 45±11 v 42±10, p=0.03 | 43±7 v 39±6, p=0.003 | 48±14v45±14, p=0.3 |
| Gender  (male) | 62 v 55%, p=0.2 | 66v57%,  p=0.3 | 57v52%,  p=0.7 | 34v42%,  p=0.3 | 32v39,  P=0.5 | 60v54%,  p=0.8 | 57v63%,  p=0.3 | 58v69,  p=0.2 | 54v56%,  p=0.8 |
| VSD | 38 v 27%, p=0.1 | 28v11%, p=0.03 | 51v58%,  p=0.5 | 30v36%,  p=0.4 | 29v20,  p=0.3 | 30v59%, p=0.03 | 37v31%,  p=0.4 | 30v14%, p=0.02 | 52v54%,  p=0.8 |
| PS | 20v23%,  p=0.6 | 13v6%,  p=0.18 | 32v52%, p=0.08 | 13v24%,  p=0.1 | 12v10,  p=0.8 | 15v44%, p=0.02 | 21v22%,  p=0.9 | 15v6,  p=0.07 | 33v44%,  p=0.3 |
| Previous TV surgery  Previous VSD surgery  Previous conduit  Any other surgery | 6v3%,  p=0.4  20 v 17%, p=0.6  13v17%, p=0.46  38v24%,  p=0.7 | 2v0%,  p=1  8v3%,  p=0.3  5v0%,  p=0.2  27v23%,  p=0.7 | 13v10%,  p=0.8  38v42%,  p=0.8  28v45%,  p=0.1  54v55%,  p=0.9 | 8v5%,  p=0.3  12v22%, p=0.09  10v16%,  p=0.3  31v38%,  p=0.4 | 5v0,  p=0.06  7v7,  p=1  7v2,  p=0.1  27v25%,  P=0.8 | 15v11%,  p=0.7  20v44%, p=0.07  15v38%, p=0.07  40v58%,  p=0.2 | 6v4%,  p=0.6  21v18%,  p=0.6  16v13%,  p=0.7  37v36%,  p=0.9 | 2v0, p=0.5  10v3,  p=0.1  5v1%,,  p=0.4  26v 25,  p=0.9 | 14v10%,  p=0.8  54v46%,  p=0.8  35v31%,  p=0.8  58v50%,  p=0.5 |
| Age at switch |  | 2.2±2.0 v 2.3±2.0,p=0.8 |  |  | 2.2±2v2.2±, p=0.9 |  |  | 2.4±2 v 1.9±1.7, p=0.12 |  |
| Baffle stenosis or leak requiring intervention |  | 25 v 18%, p=0.3 |  |  | 22v23,  p=1 |  |  | 28 v 16%, p=0.06 |  |

Supplementary Table S5. Pulmonary hypertension subtype breakdown according to baseline variables.

| Variable | Overall  Pre, CpcPH, IpcPH | p value | TGA-AS  Pre, CpcPH, IpcPH | p value | CCTGA  Pre, CpcPH, IpcPH | p value |
| --- | --- | --- | --- | --- | --- | --- |
| Age (y) | 45,44,44y | 0.9 | 42,43,42y | 0.8 | 49,47,48y | 0.9 |
| Male (%) | 54,61,74% | 0.1 | 57, 61, 87% | 0.04 | 47, 59, 58% | 0.7 |
| VSD (%) | 49, 35, 33% | 0.2 | 44, 28, 17% | 0.04 | 58, 47, 53% | 0.7 |
| PS (%) | 20, 21, 19% | 0.9 | 9, 18, 4% | 0.2 | 37, 28, 37% | 0.7 |
| Other surgery (%) | 44, 38, 30% | 0.4 | 35, 28, 17% | 0.4 | 58, 56, 47% | 0.7 |
| TV surgery (%) | 7,7,5% | 0.9 | 0,4,0% | 0.4 | 16, 13, 11% | 0.9 |
| VSD surgery (%) | 24,19,19% | 0.7 | 9,11,4% | 0.6 | 47, 34, 37% | 0.6 |
| PS surgery (%) | 17,15,7% | 0.3 | 9,18,4% | 0.2 | 42, 28, 16% | 0.2 |
| Baffle issue |  |  | 27, 28, 17% | 0.5 |  |  |
| Age atrial switch (y) |  |  | 2.5±2.4, 2.3±2.0, 1.9±1.3 | 0.5 |  |  |

**Figure S1a. PCWP according to mPAP severity and diagnosis**.

*Median PCWP is >15mmHg if mPAP 20-40mmHg and median PCWP is >20mmHg if mPAP>40mmHg (in both groups)*


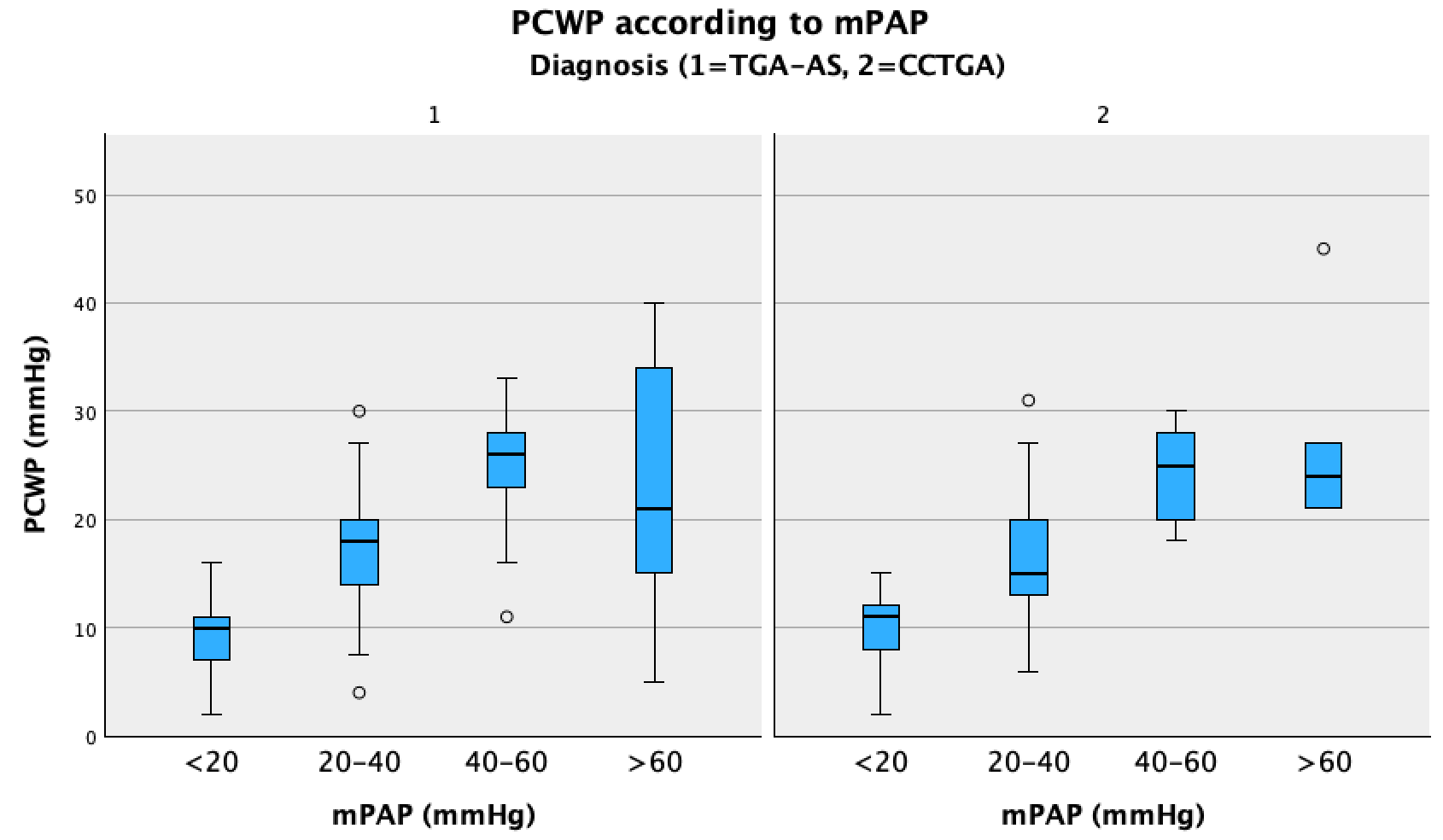


**Figure S1b. PVR according to mPAP severity and diagnosis**.

*PVR is elevated in most when mPAP>40mmHg in both groups*


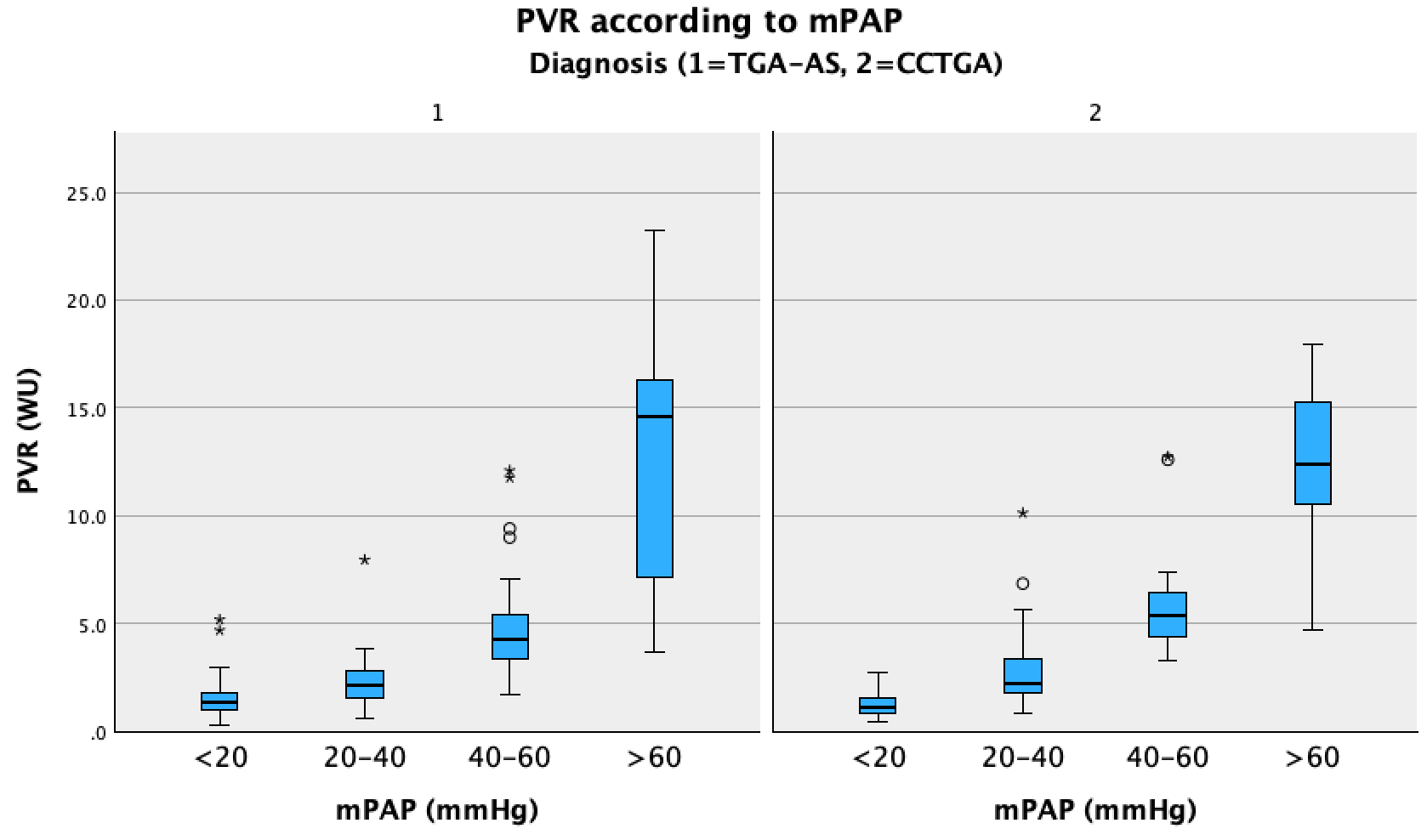

Supplement: Supplemental_Material_CLEAN [file mmc1.docx]
